# **The NIH-Moderna Vaccine:** Public Science, Private Profit, and Lessons for the Future

**DOI:** 10.1017/jme.2023.149

**Published:** 2023

**Authors:** Christopher J. Morten

**Keywords:** Vaccines, mRNA, Public-Private Partnership, Pricing, Incentives

## Abstract

This commentary highlights the scientific history of the NIH-Moderna COVID-19 vaccine and corroborates Sarpatwari’s theme of private capture of value created by the public. The commentary also identifies missteps by the Trump and Biden Administrations and offers policy recommendations: better contracts with and incentives for pharmaceutical manufacturers and a not-for-profit “public option” for pharmaceutical development.

In *Public Returns on Public Investment: Moderna’s Violation of the Social Contract*,^1^ a new article in this issue, Ameet Sarpatwari traces the extraordinary history of the NIH-Moderna COVID-19 vaccine^2^ from three perspectives: financial, legal, and political-economic. He describes a broken partnership and broken bargain between the U.S. government and Moderna. As Sarpatwari shows, Moderna received unprecedented “derisking” in the form of research funding, support for clinical trials, and an advanced market commitment; in return, the U.S. government and American people expected affordable access and some level of shared control. Moderna broke that partnership and that bargain.

This commentary responds to and builds on Sarpatwari’s article in two respects. First, it describes the history of the NIH-Moderna vaccine from a fourth perspective — scientific — which corroborates Sarpatwari’s theme of private capture of value created by the public. Second, it echoes Sarpatwari’s diagnoses and calls for reform. More specifically, this commentary identifies missteps by the Trump and Biden Administrations and offers three recommendations for the future: (1) better contracts with pharmaceutical manufacturers, (2) better incentives for those manufacturers, and (3) a not-for-profit “public option” for pharmaceutical development.

## *Moderna’s Violation of the Social Contract* Highlights Moderna’s Unprecedented “Derisking” and the Harms of High Vaccine Prices

Building on testimony Sarpatwari delivered to the United States Senate Committee on Health, Education, Labor and Pensions (HELP) in March 2023,^3^ *Moderna’s Violation of the Social Contract* describes how the U.S. government’s collaboration with Moderna to develop the wildly successful NIH-Moderna vaccine “turned the traditional model of therapeutic development on its head.”^4^ Sarpatwari’s central theme is “derisking”: the government, not Moderna, provided substantial capital and bore much of the risk.

Much of *Moderna’s Violation of the Social Contract* deftly synthesizes what others have reported about Moderna to provide a thorough accounting of the risk-reducing financial subsidies Moderna received in 2020 and 2021. Sarpatwari then builds on that synthesis with incisive analysis and timely recommendations, which I comment upon below.

Several aspects of Sarpatwari’s factual account are worth reiterating. Particularly important are two major details of Moderna’s subsidies that have escaped widespread attention. First, the U.S. government’s August 2020 procurement contract with Moderna — worth $1.5 billion, in exchange for 100 million doses of the NIH-Moderna vaccine^5^ — was apparently executed *at risk*. This means the contract seemingly committed the government to pay Moderna even if the vaccine never obtained FDA authorization or approval.^6^ As such, this first procurement contract “derisked” Moderna’s development process enormously. Second, Sarpatwari observes that, with the same contract, the U.S. government attempted to shield Moderna from any liability it might face for infringing other companies’ patents in the course of manufacturing and distributing the vaccine.^7^ In February 2023, the Department of Justice confirmed the government’s intent to provide this additional subsidy to Moderna: “the effect of the Government’s ‘authorization and consent’ is to relieve Moderna of any liability for patent infringement resulting in performance of the [August 2020 procurement contract] and to transfer to the United States any liability for the manufacture or use of the inventions claimed in [patents asserted against Moderna] resulting from the authorized and consented acts.”^8^ This liability shield may be worth tens or even hundreds of millions of dollars, as Moderna defends itself from claims of patent infringement brought by Alnylam, Arbutus, and Genevant, all of which hold patents on lipid nanoparticle technology used in mRNA delivery.^9^

Yet Moderna, and its executives and other shareholders, have managed to capture the value produced by this public-private collaboration. Moderna has now decided to quintuple its prices, from about $25 per dose to $130 — a price its executives describe as “consistent with” the full value of vaccine.^10^ Sarpatwari shows that these price increases are unjustifiable and likely to harm patients, public health, and American taxpayers for years to come.

## An Additional Scientific Perspective Corroborates Sarpatwari’s Account: Moderna Captured Valuable Science Created by the Public

I testified alongside Sarpatwari at the March 2023 hearing of the Senate HELP Committee,^11^ and I sought to present a distinct and complementary perspective on the NIH-Moderna vaccine: scientific. I attempted to answer two basic questions about the scientific history of the NIH-Moderna vaccine: What are the key features of the vaccine that make it safe, effective, stable, and otherwise valuable? Whose insights and labor created these features and this valuable product?

My research and testimony focused on three key scientific features of the NIH-Moderna vaccine:

1. The immunogen — the chemically modified coronavirus spike protein the vaccine produces once inside the body, sparking a protective immune response;
2. The modified mRNA — the stabilized, chemically modified mRNA that “encodes” the immunogen; and
3. The delivery system — the lipid nanoparticle that helps the mRNA stay stable and enter cells in the body to begin producing protein.

The choice of these three was not arbitrary; Moderna and its scientists have at points identified these three features of the NIH-Moderna vaccine as particularly important to the vaccine’s success.^12^

I found that Moderna did not invent any of these three features on its own.^13^ In fact, Moderna cannot claim to be the driving force behind *any* of these three features. Here, I provide a summary.

First, the immunogen was not invented by Moderna; it was instead invented by a publicly funded team of NIH scientists and academic collaborators working at the Scripps Research Institute and Dartmouth College,^14^ years before SARS-CoV-2 emerged. Before entering its collaboration with NIH in early 2020, Moderna had never done any work on a coronavirus vaccine, and the company relied heavily on NIH’s longstanding expertise. Writing to NIH scientist Barney Graham in January 2020, Moderna CEO Stéphane Bancel stated that Moderna would be “ready to run when you give us a sequence” of viable SARS-CoV-2 immunogen — and NIH delivered that sequence to Moderna days later.^15^ Moderna’s reliance on NIH in 2020 has not stopped the company from exaggerating its own scientific role in the years since, or from attempting, brazenly, to obtain its own patent on the immunogen sequence, omitting NIH.^16^

Second, the modified mRNA was likewise not invented by Moderna. Primary credit for the invention of the modified mRNA belongs to researchers working at the University of Pennsylvania, including Katalin Karikó and Drew Weissman.^17^ This work, and other work on modified mRNA, was again supported by NIH.^18^

Third, the delivery system was probably not invented by Moderna.^19^ The scientific history of lipid nanoparticle delivery systems is complex and somewhat murky, and details of the specific nanoparticles that Moderna uses in its vaccines are hard to come by. Yet it seems that the nanoparticles Moderna uses were invented and initially developed by researchers affiliated with the University of British Columbia and certain startup companies in British Columbia,^20^ not Moderna.

I also examined how NIH and Moderna collaborated to combine these three features (and others) into a single product, manufacturable at scale. Combining these features into a complete, validated vaccine in a matter of months was a momentous achievement in its own right. However, as I described in my testimony, again it seems the U.S. government did at least as much as Moderna.^21^ For example, NIH designed, paid for, and ran the early clinical trials on the original NIH-Moderna vaccine,^22^ and it later helped Moderna develop its first variant-specific booster shot.^23^ Moreover, Operation Warp Speed provided equipment and other support to expand Moderna’s manufacturing.^24^ Then-head of Operation Warp Speed Moncef Slaoui summed it up in late 2020: “We held Moderna by the hand on a daily basis.”^25^

Operation Warp Speed’s negotiators botched their negotiation. Back in the hectic, horrible, uncertain days of 2020, the Trump Administration failed to extract legally binding concessions from Moderna in exchange for the unprecedented derisking it gave the company. These concessions might have included contractual commitments to fair pricing, data sharing, and global access to mRNA vaccines; shared control of intellectual property, manufacturing data, and Moderna’s scientific agenda; or perhaps some of the more quotidian concessions given to major private investors, such as shares and voting board seats.

Of course, Moderna’s scientists and engineers made significant contributions of their own to the NIH-Moderna vaccine. These include the vaccine’s “stop codon,” a commercial-scale manufacturing process, and improvement of the modified mRNA.^26^ Moderna’s scientists, engineers, and other employees deserve celebration alongside NIH’s and their academic collaborators.

Overall, however, the scientific history undermines Moderna’s executives’ efforts to claim the full value of the NIH-Moderna vaccine for the company.

## Lessons and Recommendations for the Future

Today, Moderna controls the factories, key intellectual property and tacit knowledge, the regulatory filings, and the scientific agenda for future mRNA research. The government finds itself transformed from senior partner in its partnership with Moderna to a helpless customer.

The shrunken power of the government is arguably best highlighted by a July 2023 letter that Xavier Becerra, Secretary of Health & Human Services, sent to Moderna and Pfizer.^27^ Becerra essentially begged the companies not to increase vaccine prices: “Price gouging behavior takes advantage of the trust the American people have placed in you through the COVID-19 response.”^28^

Neither Moderna nor Pfizer has given any indication they will lower prices. In fact, COVID-19 vaccine prices are likely to rise for years to come, according to the industry’s standard playbook.^29^ As Sarpatwari explains, the likely consequences of high prices are grim: increased inequity and public health costs, and more illness and death from COVID-19.^30^

All this begs a critical question: If the American public created the lion’s share of the value of the NIH-Moderna vaccine, why is the public now left with so little?

A simple answer to the question is that Operation Warp Speed’s negotiators botched their negotiation. Back in the hectic, horrible, uncertain days of 2020, the Trump Administration failed to extract legally binding concessions from Moderna in exchange for the unprecedented derisking it gave the company. These concessions might have included contractual commitments to fair pricing, data sharing, and global access to mRNA vaccines; shared control of intellectual property, manufacturing data, and Moderna’s scientific agenda; or perhaps some of the more quotidian concessions given to major private investors, such as shares and voting board seats.^31^

A more nuanced answer acknowledges that the Biden Administration missed opportunities of its own to renegotiate for more. For example, in early 2023, Moderna announced that it would pay NIH hundreds of millions of dollars for a license to NIH’s key patent on the immunogen in the NIH-Moderna vaccine; rather than collect this revenue, Biden and NIH could have used those hundreds of millions in financial leverage to extract concessions from the company on pricing and access.^32^ Better yet, Biden could have used the Defense Production Act (DPA) —a federal statute enacted in 1950 that gives the U.S. President broad and unilateral authority to protect the national defense.^33^ In 2021, as vaccine supplies were scarce and global manufacturing capacity sat idle, Biden could have used the DPA to compel Moderna to share valuable information on mRNA vaccine manufacturing with the World Health Organization, thereby expanding supplies and access, limiting the spread of new variants to the United States, saving lives here and globally, and catalyzing new research.^34^

Another critical question: what to do now? Sarpatwari argues that our government could still act to urge Moderna to lower its prices, and/or mitigate the harms of high prices. He proposes increased Congressional pressure on the company; continuation of bulk purchases of vaccines; and Congressional authorization to the Centers for Medicare and Medicaid Services (CMS) to negotiate prices.^35^ I agree.

Sarpatwari looks ahead to future public health emergencies. In *Moderna’s Violation of the Social Contract* and elsewhere,^36^ Sarpatwari argues for a new law and policy framework for public-private partnerships between the U.S. government and pharmaceutical companies. For Sarpatwari, an essential component of this new framework will be explicit, enforceable contractual provisions in government and industry partnerships to ensure affordable prices.^37^ Again, I agree.

In separate writing,^38^ I elaborate on long-term lessons I think we should take from the successes and failures of Operation Warp Speed. In brief, I believe our government should both restructure its legal arrangements with the pharmaceutical industry and, simultaneously, explore the possibility of discovering, developing, manufacturing, and developing pharmaceutical products without relying on that rapacious industry.

The government can and should restructure legal arrangements with industry by contracting better—with fair pricing and global access provisions, commitments to data sharing, government representatives on corporate boards, and so on as noted above—and by experimenting with alternative incentive structures that entice industry but leave greater control in the hands of the public. Such incentive structures include innovation prizes^39^ and “government-owned, contractor-operated” (GOCO) partnership models.^40^ Government-set innovation prizes would align R&D efforts with the greatest public health needs and would “de-link” financial incentives for industry from prices, meaning industry could be guaranteed a healthy financial reward for useful innovation while also guaranteeing low prices for patients.^41^ The GOCO model—already widely used by the U.S. Departments of Defense and Energy—pays companies to operate R&D and manufacturing facilities but retains public ownership of those facilities.^42^

Simultaneously, the government can and should foster a genuine alternative to the profit-hungry pharmaceutical industry. We need a “public option” comprising government-owned, government-operated pharmaceutical research and development and manufacturing, and focused not on profit but on public health and the advancement of human knowledge.^43^

The opportunity for reform is ripe. Congress and the Biden Administration have announced major new investments and initiatives in research and development (such as Project NextGen (which will develop new technologies against SARS-CoV-2)^44^ and ARPA-H (which expands NIH’s ability to do high-risk, high-reward research)^45^). Congress is considering more (such as a reauthorized and expanded Pandemic and All-Hazards Preparedness Act (which invests billions in pandemic preparedness)^46^). Promisingly, the Biden Administration just included an explicit, enforceable reasonable pricing provision in a new $326 million investment contract with Regeneron struck as part of Project NextGen.^47^

The stakes are high. Debates over not just pharma and health care but also housing, transportation, energy, response to climate change, and more all turn on fundamental questions of the legal structure of public investment in new technologies.^48^ *Moderna’s Violation of the Social Contract* illuminates some of the weaknesses of the current paradigm of public-private partnership and points us to a new one.

## Note

I thank Austin DeRamus for invaluable research assistance with this piece. Relationships: Unpaid (pro bono) legal representation of PrEP4All, which has advocated for broader, more affordable access to mRNA-based vaccines. Member of the Advisory Board of the Collaboration for Regulatory Rigor, Integrity & Transparency at Yale University
